# Enhancing the Quality of Single Cone Obturation Using Hydroxyapatite Precursor Grafted Nanocomplex for Dentine Conditioning: An In Vitro Study

**DOI:** 10.1111/iej.70034

**Published:** 2025-09-12

**Authors:** Eissa Sameer Bunashi, Mingxin Hu, Angeline Hui Cheng Lee, Chengfei Zhang, Anil Kishen, Jeffrey Wen Wei Chang

**Affiliations:** ^1^ Restorative Dental Sciences, Endodontics, Faculty of Dentistry The University of Hong Kong Hong Kong P. R. China; ^2^ Kishen Lab, Faculty of Dentistry University of Toronto Toronto Ontario Canada

**Keywords:** chitosan‐hydroxyapatite precursor nanocomplexes, conditioning, micro‐computed tomography, single cone obturation, voids

## Abstract

**Aim:**

This study aimed to quantify the effect of chitosan‐hydroxyapatite precursor nanocomplexes (C‐HA) on void reduction in the root canal system and isthmus regions, characterise void distribution patterns and assess sealer‐dentin interface adaptation post‐conditioning.

**Methodology:**

Vertucci type II and Yin type IV mesial root canals from 24 extracted mandibular first molars were randomly divided into two groups (*n* = 12): (1) C‐HA conditioned and (2) control (deionised water). All canals underwent standardised preparation using ProTaper Next files with 3% NaOCl irrigation (2 mL per file change) and final irrigation with 17% EDTA (5 mL, sonic activation) followed by 3% NaOCl (5 mL) and a final deionised water rinse (5 mL). Before obturation, the C‐HA group received 5 min of C‐HA solution (2 mg/mL) treatment with active agitation by ProTaper Next X2 gutta‐percha cone, while the control group received equivalent water treatment. Canals were obturated using the single‐cone technique with Ceraseal. High‐resolution Micro‐CT (8.6 μm voxel size) quantified void percentages in the entire canal system and isthmus regions, with analysis stratified by coronal, middle and apical thirds. Selected specimens underwent SEM evaluation of interfacial adaptation. Statistical analysis used the independent samples *t*‐test, two‐way ANOVA with Tukey's post hoc tests (*α* = 0.05), and Fisher's exact test.

**Results:**

No significant differences related to the canal/isthmus pre‐treatment volumes and isthmus characteristics were noted between the two groups. Micro‐CT analysis revealed significantly lower void percentages in C‐HA‐treated canals compared to controls (9.21% vs. 19.01% in the entire canal system and 19.07% vs. 55.65% in the isthmus regions, *p* < 0.001). Analysis of the void distribution patterns further demonstrated statistically significant differences between the groups in the cervical and middle root thirds (*p* < 0.05). SEM evaluation showed continuous, gap‐free interfaces with bioactive mineralization in C‐HA‐treated canals, contrasting with discontinuous adaptation and sparse precipitates in controls.

**Conclusion:**

C‐HA dentine conditioning significantly improved obturation quality with Ceraseal bioceramic sealer by reducing voids in isthmus regions and enhancing sealer‐dentin interfacial adaptation.

## 1 Introduction

The long‐term success of root canal treatment heavily relies on the elimination of biofilms through comprehensive chemomechanical debridement and the formation of a hermetic, three‐dimensional obturation within the root canal system. However, achieving optimal interfacial bonding between endodontic sealer and radicular dentine remains a significant clinical challenge. This is attributable to the intricate microstructure of dentine: a hierarchical biocomposite composed of carbonated hydroxyapatite (60–70 vol%), type I collagen (20 vol%), and non‐collagenous proteins (Goldberg et al. 2011). While conventional irrigants such as sodium hypochlorite (NaOCl) and ethylenediaminetetraacetic acid (EDTA) are indispensable for dissolving organic pulp remnants and removing the smear layer, they inadvertently alter the structural integrity of dentine. NaOCl oxidises and degrades collagen fibrils, leading to their denaturation and reduced biomechanical resistance, whereas EDTA chelates the calcium ions and demineralises peritubular dentine, increasing tubular diameter and surface roughness (Ferrari et al. 2004; Ballal et al. 2013; Gu et al. 2017). These dual effects diminish dentine surface free energy and hydrophilicity, impairing the penetration of sealers into dentinal tubules, thus increasing the risks of interfacial gaps, microleakage and recurrent infection (Kishen 2006).

The quality of root canal obturation is determined not only by its radiographic length, density and taper but also by how effectively it seals the entire root canal system, including anatomical complexities such as isthmuses, fins and lateral canals (Schilder 2006; Ricucci and Siqueira 2010). The sealer's ability to flow into these irregular spaces is crucial for maximising the seal and entombing residual microorganisms that may persist despite thorough cleaning and shaping. Previous studies have assessed various methods to evaluate sealer penetration into dentinal tubules and anatomical complexities (Weis et al. 2004; Iglecias et al. 2017; Zhang et al. 2021; Ma and Liang 2025). This parameter becomes even more important in sealer‐based obturation techniques combined with the single‐cone method, as the quality of isthmus penetration depends primarily on the sealer's properties in the absence of condensation pressure. Notably, studies have demonstrated that the single‐cone technique resulted in significantly higher void volumes in isthmus areas compared to other obturation methods (Zhang et al. 2021; Ma and Liang 2025). These findings highlight the necessity for developing innovative strategies to address these limitations in single‐cone obturation within modern endodontic practice.

Bioactive conditioning agents have been investigated as a solution to improve the obturation quality by modifying dentine surfaces and maintaining their structural and biomechanical integrity. Among the various biomaterials explored, chitosan‐hydroxyapatite precursor nanocomplexes (C‐HA) represent a novel and promising alternative due to their unique physicochemical properties and bioactive potential. C‐HA nanocomplexes, with an average diameter of less than 50 nm, were synthesised through the integration of carboxymethyl chitosan and amorphous calcium phosphate (Chen et al. 2015). Carboxymethyl chitosan exhibits antibacterial and hydrophilic properties (Gonçalves et al. 2017; Kalliola et al. 2018), while amorphous calcium phosphate serves as a reactive precursor for remineralization (Reynolds 2008). This synergistic combination enabled C‐HA with multifunctional capabilities. Previous studies have demonstrated that C‐HA treatment significantly enhances the ultimate tensile strength of dentine, reduces post‐obturation microbial load and improves resistance to fracture (Hashmi, Zhang, and Kishen 2019; Del Carpio‐Perochena et al. 2022). Furthermore, the nanocomplexes greatly increased surface wettability, which facilitated deeper penetration of tricalcium silicate (TCS)‐based sealers into dentinal tubules (Hashmi, Sodhi, and Kishen 2019; Hashmi, Zhang, and Kishen 2019). Although the smear layer removal efficacy of the C‐HA complex requires further validation, its chitosan component has been proven effective in smear layer removal with minimal dentine erosion (Silva et al. 2013), suggesting potential for similar effects in the C‐HA complex.

This study aimed to address these research gaps by using Micro‐computed tomography (Micro‐CT) and scanning electron microscopy (SEM) to (1) quantify void percentage and distribution patterns, (2) assess sealer penetration efficacy in isthmus regions and (3) evaluate interfacial adaptation after C‐HA conditioning in the single cone obturation system. The null hypothesis was that C‐HA dentine conditioning does not reduce void percentage in the entire root canal system and isthmus regions or improve sealer‐dentin interfacial adaptation compared to untreated controls. The findings provided important clinically relevant evidence to optimise bioactive single‐cone obturation protocols, particularly when managing anatomically complex root canal systems.

## 2 Materials and Methods

**FIGURE 1:**
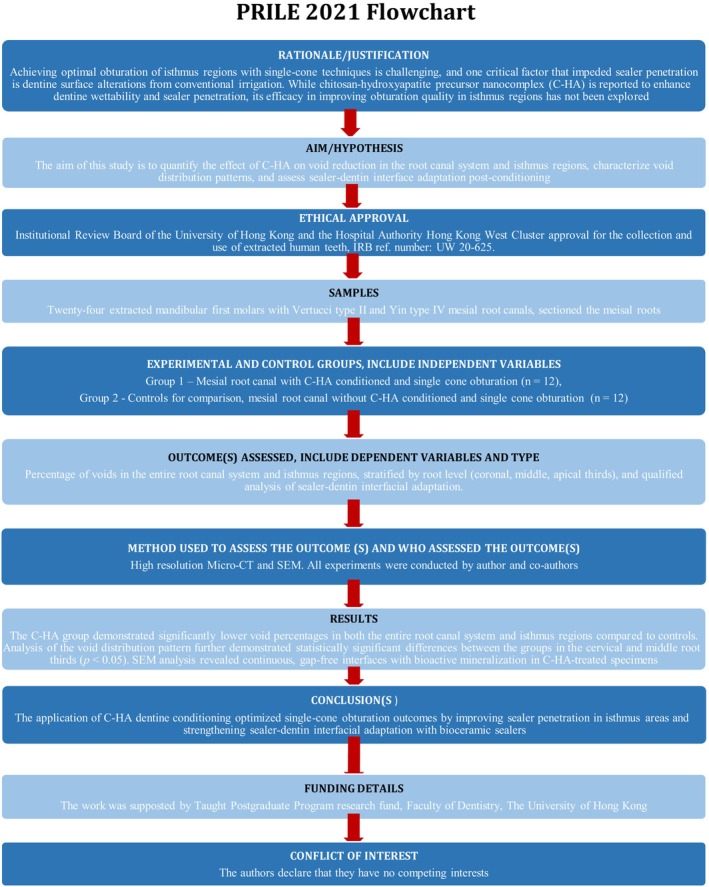
PRILE 2021 flowchart illustrating the steps involved in this study.

**FIGURE 2:**
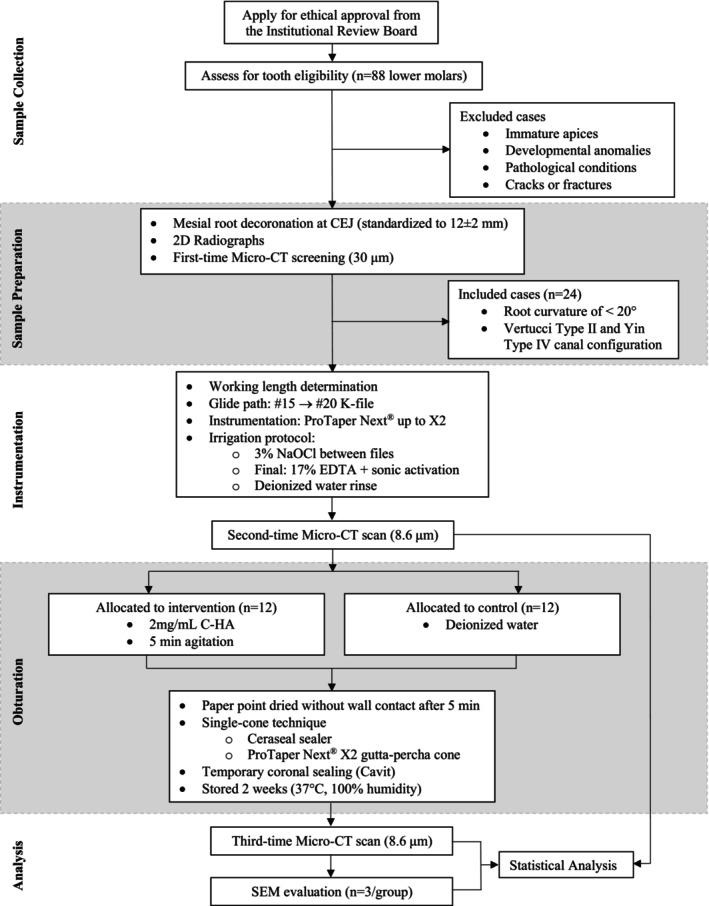
Schematic representation of the experimental workflow.

The manuscript of this study has been written according to Preferred Reporting Items for Laboratory Studies in Endodontology (PRILE) 2021 guidelines (Nagendrababu et al. 2021) (Figure 1). Ethical approval was obtained from the Institutional Review Board of the University of Hong Kong and the Hospital Authority Hong Kong West Cluster (Reference No: UW 20‐625) for the collection and use of extracted human teeth. All teeth were sourced from an anonymized collection of freshly extracted teeth at a dental teaching hospital, and Informed Consents were obtained from all the volunteers before the tooth collection. In accordance with ethical standards and to protect patient privacy, no demographic information (including age or gender) was collected during the specimen acquisition. The complete experimental workflow was presented schematically in Figure 2.

### 2.1 Sample Selection and Preparation

Eighty‐eight extracted human mandibular molars were collected and preserved in 0.1% thymol solution at 4°C. All specimens were disinfected through immersion in 6% sodium hypochlorite (Clorox, Oakland, CA, USA) for 5 min, supplemented by ultrasonic scaling (Piezon EMS SA, Nyon, Switzerland) to remove residual soft and hard root‐adherent tissues. Teeth were selected based on the following criteria: intact structure, fully developed roots, absence of developmental anomalies, pathological conditions, cracks, or fractures. The selected teeth were then decoronated at the cementoenamel junction using a custom‐made low‐speed saw (1500 rpm) under water cooling. The mesial roots were isolated and standardised to a length of 12 ± 2 mm. Roots exhibiting curvature exceeding 20°, as confirmed by periapical radiograph (Romexis, Planmeca, Helsinki, Finland) in both buccolingual and mesiodistal directions, were excluded from this study (Schneider 1971). Three‐dimensional analysis was performed using Micro‐CT (Skyscan 1172, Bruker‐microCT, Kontich, Belgium). The sample size was calculated using G*Power 3.1 (Heinrich Heine University, Düsseldorf, Germany) for a two‐tailed *t*‐test comparing independent groups. Based on the previous study (Endal et al. 2011), an effect size of 1.21 was derived, with *α* = 0.1 and *β* = 0.20 (80% power). The analysis indicated a minimum requirement of 11 specimens per group. To avoid potential technical variations, 12 specimens per group were included. Based on Micro‐CT data, 24 mesial roots with Vertucci Type II canal configuration (Vertucci 1984) and Yin Type IV non‐boundary isthmuses (Yin et al. 2021) were selected for further experiments. A detailed assessment of isthmus characteristics after root canal preparation was performed using CTAn software (v1.20.3.0, Bruker‐microCT) described in the following section.

### 2.2 Root Canal Preparation

The working length determination was performed using a size 10 K‐FlexoFile (Dentsply Maillefer, Ballaigues, Switzerland). The instrument was gently advanced until visible at the apical foramen, after which 1 mm was subtracted from this measurement to establish the final working length. To accurately simulate clinical conditions, a closed‐end system was created by sealing each apical foramen with wax before initiating the instrumentation procedures.

All procedures were conducted by a single endodontist to ensure procedural consistency and minimise operator variability. Glide paths were established using sequential #15 and #20 K‐FlexoFiles, after which both mesial canals were instrumented with ProTaper Next rotary nickel‐titanium files (Dentsply Maillefer) up to size X2 using an X‐smart Plus endodontic motor (Dentsply Maillefer) operating at 300 rpm with 2 Ncm torque according to manufacturer specifications. During instrumentation, canals were irrigated at each change of files with 2 mL aliquots of 3% NaOCl delivered via a 30‐gauge NaviTip needle (Ultradent Products Inc., South Jordan, UT, USA) mounted on a 5 mL syringe (Terumo Europe, Leuven, Belgium), maintaining the needle tip 2 mm short of working length. Upon completion of instrumentation, a final irrigation protocol was performed, consisting of the following steps: (1) 5 mL of 17% EDTA for 60 s, with sonic activation using a 15/02 tip (EQ‐S, Meta Biomed Co., Cheongju‐si, Chungcheongbuk‐do, Korea). (2) 5 mL of 3% NaOCl as a final disinfectant rinse. (3) 5 mL of deionised water to remove any residual irrigants. All prepared specimens were then labelled, wrapped in moistened gauze, and stored in sealed containers at ambient temperature until subsequent Micro‐CT analysis.

**FIGURE 3:**
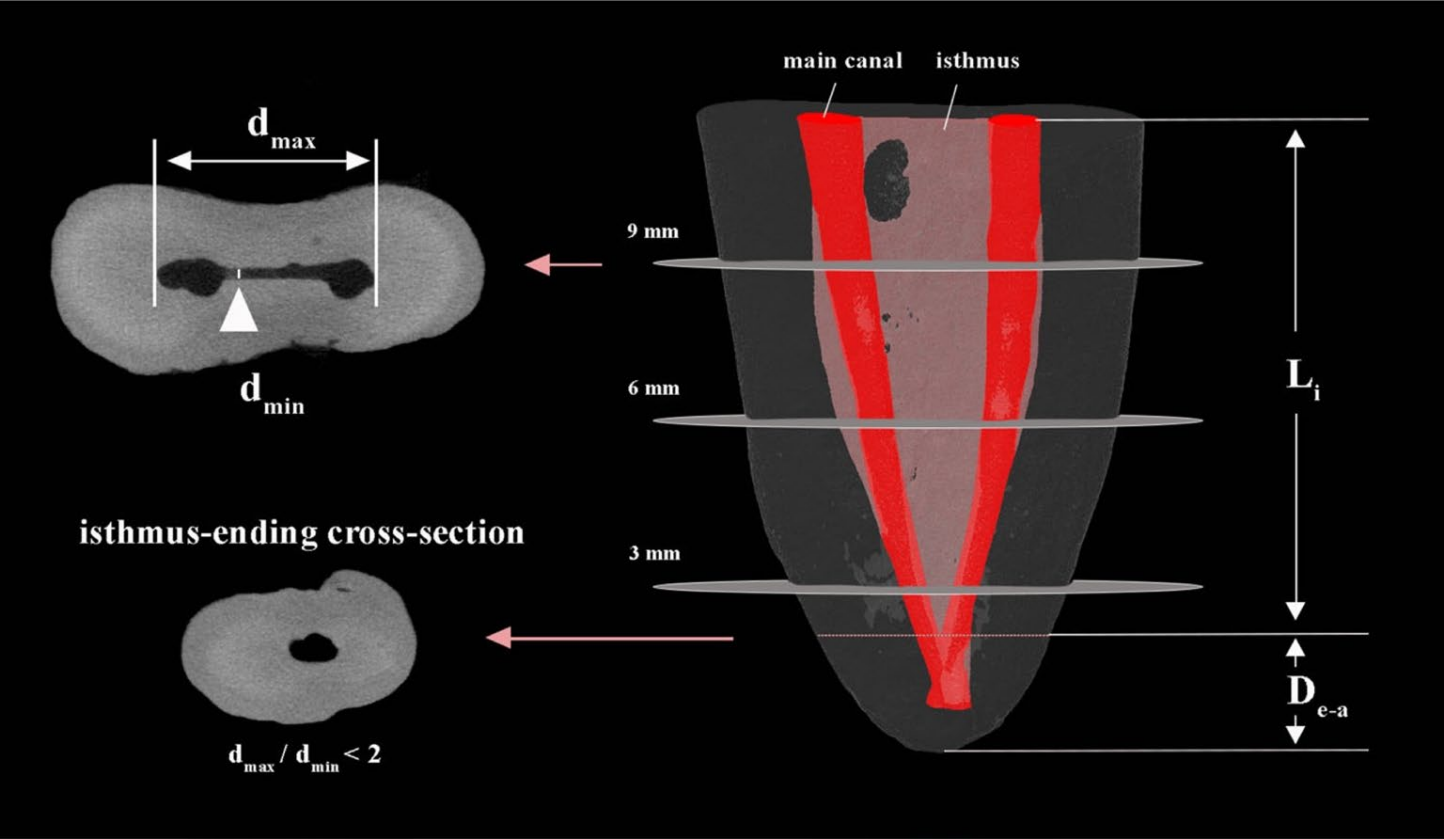
Micro‐CT analysis of isthmus morphology. The main root canals and isthmuses were digitally reconstructed in red and pink, respectively. Cross‐sectional measurements were taken at three levels: apical (3 mm from apex), middle (6 mm) and coronal (9 mm). *d*_max_: the maximum diameter of the isthmus in the same cross‐sections. *d*_min_: the minimum diameter of the isthmus in the same cross‐section. The isthmus‐ending cross‐section was defined as the first cross‐section where the *d*_max_/*d*_min_ < 2. *D*_e‐a_: the distance from the isthmus ending cross‐section to the anatomical apex. *L*_i_: the axially continuing length of the isthmus.

By using CTAn (v1.20.3.0, Bruker‐microCT), the post‐prepared canal and isthmus characteristics were evaluated cross‐sectionally (Yin et al. 2021), including: (1) volumetric quantification of root canals and isthmuses within the entire root canal system, stratified by anatomical level (coronal, middle and apical thirds). (2) Measurement of maximum (*d*_max_) and minimum (*d*_min_) diameters at three cross‐sections: apical portion (3 mm from the anatomical apex), middle portion (6 mm from the anatomical apex), coronal portion (9 mm from the anatomical apex), thus assessing the bucco‐lingual and mesio‐distal extension of the isthmus (Ma and Liang 2025). (3) Classification of isthmus type at each cross‐section according to the Hsu and Kim (1997) to evaluate morphological variations. (4) Determination of isthmus length (L_i_), defined as the axial distance from the coronal obturation endpoint to the isthmus‐ending cross‐section (a maximum‐to‐minimum diameter ratio < 2). (5) Measurement of distance from the isthmus‐ending cross‐section to the anatomical apex (*D*_e−a_) to assess apical termination of the isthmus (Figure 3).

### 2.3 Root Canal Obturation

The prepared mesial roots were randomly allocated into two experimental groups (*n* = 12 per group) through stratified randomization: an experimental group treated with C‐HA and a negative control group receiving no C‐HA treatment. Both groups were obturated following the standardised single‐cone technique with CeraSeal bioceramic sealer (Meta Biomed Co.).

For the experimental group, a 2 mg/mL C‐HA solution was prepared by combining C‐HA powder (Kishen Laboratory, University of Toronto, Canada) with deionised water (Hashmi, Zhang, and Kishen 2019). Each root canal system received 1 mL of the C‐HA solution, delivered via a 30‐gauge NaviTip irrigation needle (Ultradent Products Inc.) placed 2–3 mm short of the working length. The solution was then actively agitated for 5 min using a ProTaper Next X2 gutta‐percha cone (Dentsply Maillefer) with a standardised agitation protocol of three vertical strokes per second (Hashmi, Zhang, and Kishen 2019). After the treatment period, the complete removal of the C‐HA solution was confirmed. The root canal was then carefully dried using sterile fine paper points (Meta Biomed Co.), ensuring minimal contact with the canal wall up to the middle third. The control group received the same procedural protocol, with 1 mL of deionised water substituted for the C‐HA solution.

The CeraSeal bioceramic sealer was injected into each canal using the manufacturer's provided applicator tip, with careful termination approximately 4 mm short of the established working length. A ProTaper Next X2 master gutta‐percha cone, coated with sealer, was then gently placed to the full working length using controlled pumping motions. This technique consisted of applying gentle apical pressure followed by slight withdrawal approximately 0.5–1 mm, repeated in a deliberate, rhythmic manner (one cycle per second for 5–10 s) to facilitate optimal sealer distribution along the entire canal wall surface while minimising void formation.

Following cone placement, the coronal excess was cleanly removed using the Calamus Dual 3D obturation system (Dentsply Sirona, Charlotte, NC, USA). Immediate vertical compaction was performed with a #1 Buchanan hand plugger (SybronEndo, Orange, CA, USA) to enhance apical seal integrity. Temporary coronal seals were then established using Cavit provisional restorative material (3M ESPE, St. Paul, MN, USA). All obturated specimens were subsequently transferred to a controlled environmental chamber (37°C ± 1°C, 100% relative humidity) for a standardised 14‐day incubation period to ensure complete sealer setting before the third Micro‐CT evaluation (Kim et al. 2021).

### 2.4 Micro‐CT Scanning and Image Analysis

Micro‐CT imaging was performed at three experimental stages using standardised protocols. Initial baseline scans were acquired prior to instrumentation using a SkyScan 1172 system (Bruker‐microCT) with the following acquisition parameters: 80 kV, 100 mA, 180° rotation with a 1° rotation step, isotropic voxel size of 30 μm and a 0.5 mm Al filter. Following canal preparation and obturation procedures, high‐resolution scans were obtained using a SkyScan 1076 scanner (Bruker‐microCT) with optimised imaging parameters: 88 kV, 110 μA, 360° rotation with a 0.6° rotation step, isotropic pixel size of 8.6 μm and a 1 mm Al filter, and a 2300 ms exposure time without frame averaging.

Raw scan data were reconstructed in NRecon software (v1.7.4.6, Bruker‐microCT). Reconstruction parameters included 40% beam hardening correction, 10% ring artefact reduction, and minimal image smoothing (level 1) to preserve anatomical detail. Following reconstruction, datasets were spatially normalised in DataViewer (v1.5.6.2, Bruker‐microCT) to establish three‐dimensional coordinates for comparative analysis. Volumetric quantification was conducted in CTAn (v1.20.3.0, Bruker‐microCT) using calibrated grayscale thresholds: 0 to 80 for canal space identification and 115–255 for obturation material. The intercanal isthmus region, characterised as the narrow connective tissue between mesial canals, was isolated by segmentation tools for separate analysis.

Quantitative assessment of obturation quality was performed using standardised volumetric calculations.

#### 2.4.1 Whole Canal System Analysis

The total void volume (Voids_tvol_) = Post‐instrumentation canal volume (Canal_tvol_) – Filling materials volume (Filling_tvol_). Percentage void volume (%Voids_tvol_) was calculated using the following formula (Iglecias et al. 2017):

$$\%{\text{Voids}}_{\text{tvol}}=\frac{{\text{Voids}}_{\text{tvol}}}{{\text{Canal}}_{\text{tvol}}}\times 100\%$$

#### 2.4.2 Isthmus‐Specific Analysis

Isthmus void volume (Voids_ivol_) = Post‐instrumentation isthmus volume (Canal_ivol_) − Isthmus filling volume (Filling_ivol_). Percentage void volume in isthmus regions (%Voids_ivol_) was derived from the following formula:

$$\%{\text{Voids}}_{\text{ivol}}=\frac{{\text{Voids}}_{\text{ivol}}}{{\text{Canal}}_{\text{ivol}}}\times 100\%$$

All volumetric measurements were conducted separately for the coronal, middle and apical canal thirds to evaluate the distribution of the voids.

### 2.5 SEM Evaluation

For interfacial characterisation, three representative specimens from each experimental group were randomly selected. The samples were sectioned horizontally at 4 mm from the apex using a low‐speed 0.3 mm saw (Isomet 1000, Buehler, USA) operating at 1500 rpm under continuous water irrigation to prevent thermal damage. Following sectioning, specimens underwent standardised dehydration in a desiccator chamber maintained at 25°C for 24 h to ensure complete moisture removal and were sputter‐coated with 80% platinum and 20% palladium coating through a magnetron ion sputter device (MSP‐2S, IXRF Systems, Austin, TX, USA). The prepared samples were then examined under SEM (SU1510, Hitachi High‐Technologies, Tokyo, Japan) at sequentially increasing magnifications.

### 2.6 Statistical Analysis

All data were presented as the mean ± standard deviation. Normality testing was conducted using both Kolmogorov–Smirnov and Shapiro–Wilk tests. All statistical comparisons were performed in GraphPad Prism (v6.0, IBM, USA) using the independent samples *t*‐test, two‐way ANOVA with Tukey's *post hoc* analysis for multiple comparisons, and Fisher's exact test. Statistical significance was established at *p* < 0.05.

## 3 Results

### 3.1 Micro‐CT Evaluation

**FIGURE 4:**
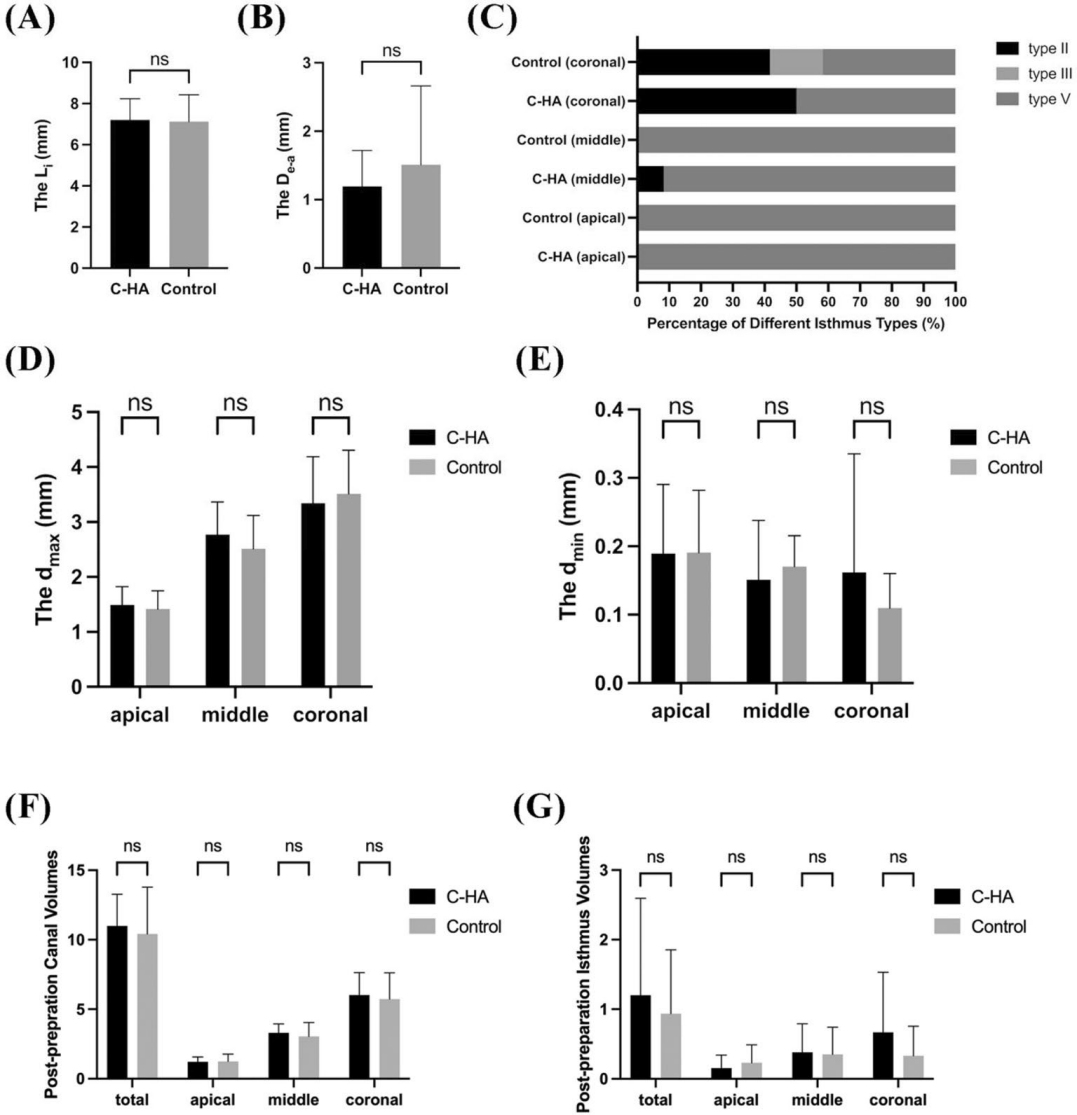
Quantitative analysis of isthmus characteristics. (A) Isthmus length (*L*_i_) comparison between groups. (B) Distance from isthmus‐ending cross‐section to radiographic apex (*D*_e‐a_) by group. (C) Distribution of isthmus types across root levels (Fisher's exact test, *p* > 0.05). (D, E) Maximum (*d*_max_) and minimum isthmus diameter (*d*_min_) at apical (3 mm), middle (6 mm) and coronal (9 mm) cross‐sections. Post‐preparation volumes in (F) complete canal systems and (G) isthmus regions, stratified by root level. Data represent mean ± standard deviation; ns indicates non‐significant intergroup differences (*p* > 0.05).

Representative two‐dimensional axial cross‐sections of mesial roots, before and after instrumentation, from both the control and C‐HA‐treated groups are presented in the Figure S1. Quantitative evaluation demonstrated comparable post‐preparation canal and isthmus space between C‐HA treatment and control groups, with no significant differences observed in volumetric measurements across all root levels (*p* > 0.05) (Figure 4F,G). The analysis also revealed equivalent isthmus dimensions, including isthmus length (*L*_i_: 7.21 ± 1.03 mm vs. 7.13 ± 1.31 mm, *p* > 0.05, Figure 4A), apical termination distance (*D*_e‐a_: 1.19 ± 0.52 mm vs. 1.51 ± 1.15 mm, *p* > 0.05, Figure 4B), and cross‐sectional diameters (*d*_max_ and *d*_min_) at any anatomical level (apical, middle, or coronal) (*p* > 0.05, Figure 4D,E). Both groups consistently showed Type V isthmus morphology in the apical regions, with no significant differences between them (*p* > 0.05, Figure 4C). Although middle and coronal regions presented more varied isthmus types (including Type II and III), the pattern of variation was statistically similar between the C‐HA treatment and control groups (*p* > 0.05, Figure 4C). These findings confirmed successful randomization and established a comparable baseline for subsequent obturation quality assessment.

**FIGURE 5:**
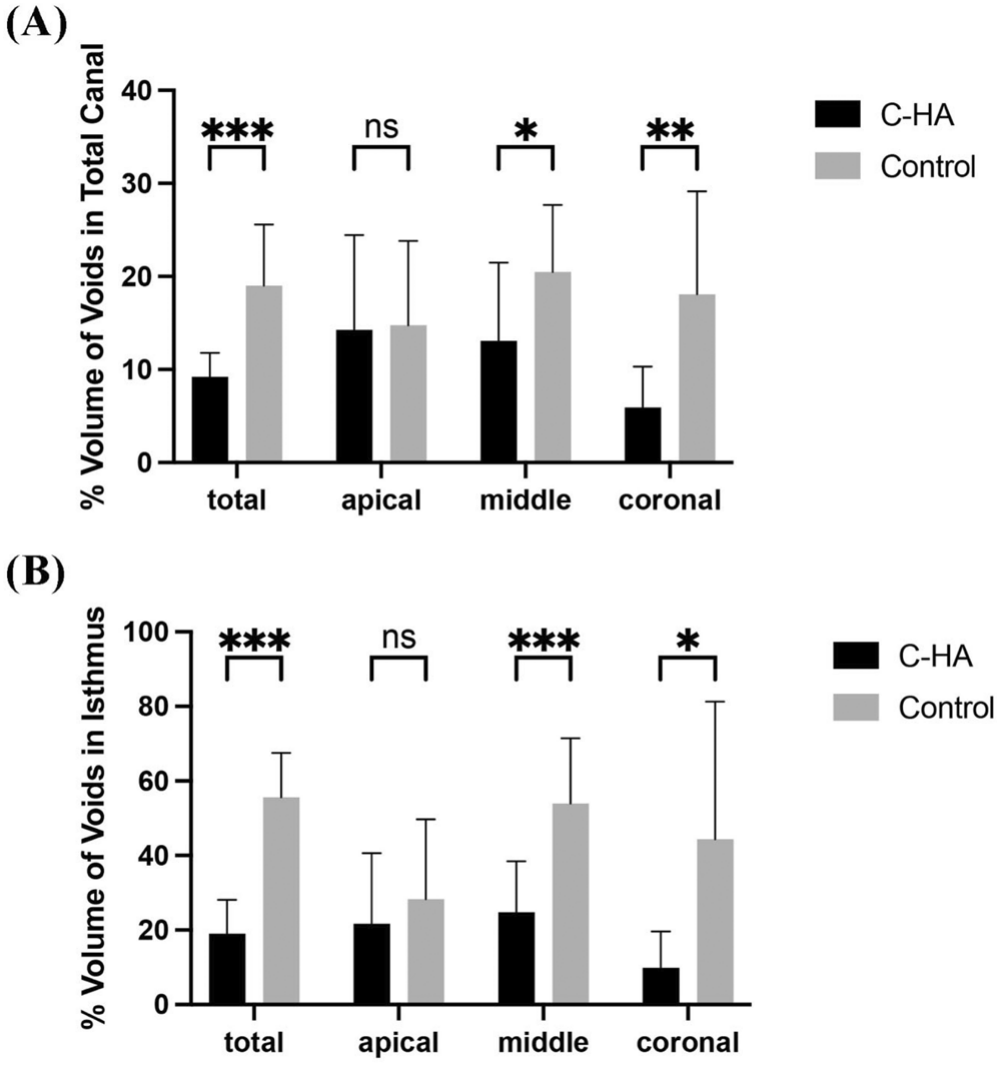
Percentage void volume distribution across root canal levels: (A) entire canal system and (B) isthmus regions following single‐cone obturation, stratified by coronal, middle and apical thirds. Data presented as mean ± SD. ns, no significant difference; *p < 0.05, **p < 0.01 and ***p < 0.001.

Quantitative Micro‐CT analysis revealed significantly improved obturation quality in the C‐HA treatment group, demonstrating a markedly lower mean total void percentage (9.21% ± 2.59%) compared to controls (19.01% ± 6.58%; *p* < 0.001, Figure 5A). Analysis of the void distribution pattern further demonstrated statistically significant differences between the groups in the cervical and middle root thirds (*p* < 0.05), whereas no significant difference was observed in the apical region (*p* > 0.05). The isthmus regions, present throughout the entire root canal system, accounted for the majority of void formation. C‐HA treatment significantly reduced void percentages in these regions (19.07% ± 9.07%) compared to controls (55.65% ± 11.93%; *p* < 0.001, Figure 5B). This reduction was statistically significant in the coronal and middle thirds of the isthmus (*p* < 0.05), while apical isthmus regions showed comparable void percentages between groups (*p* > 0.05). These findings demonstrated that C‐HA effectively reduced void formation in more accessible coronal and middle isthmus regions, while its effect on apical isthmus regions appeared less pronounced.

**FIGURE 6:**
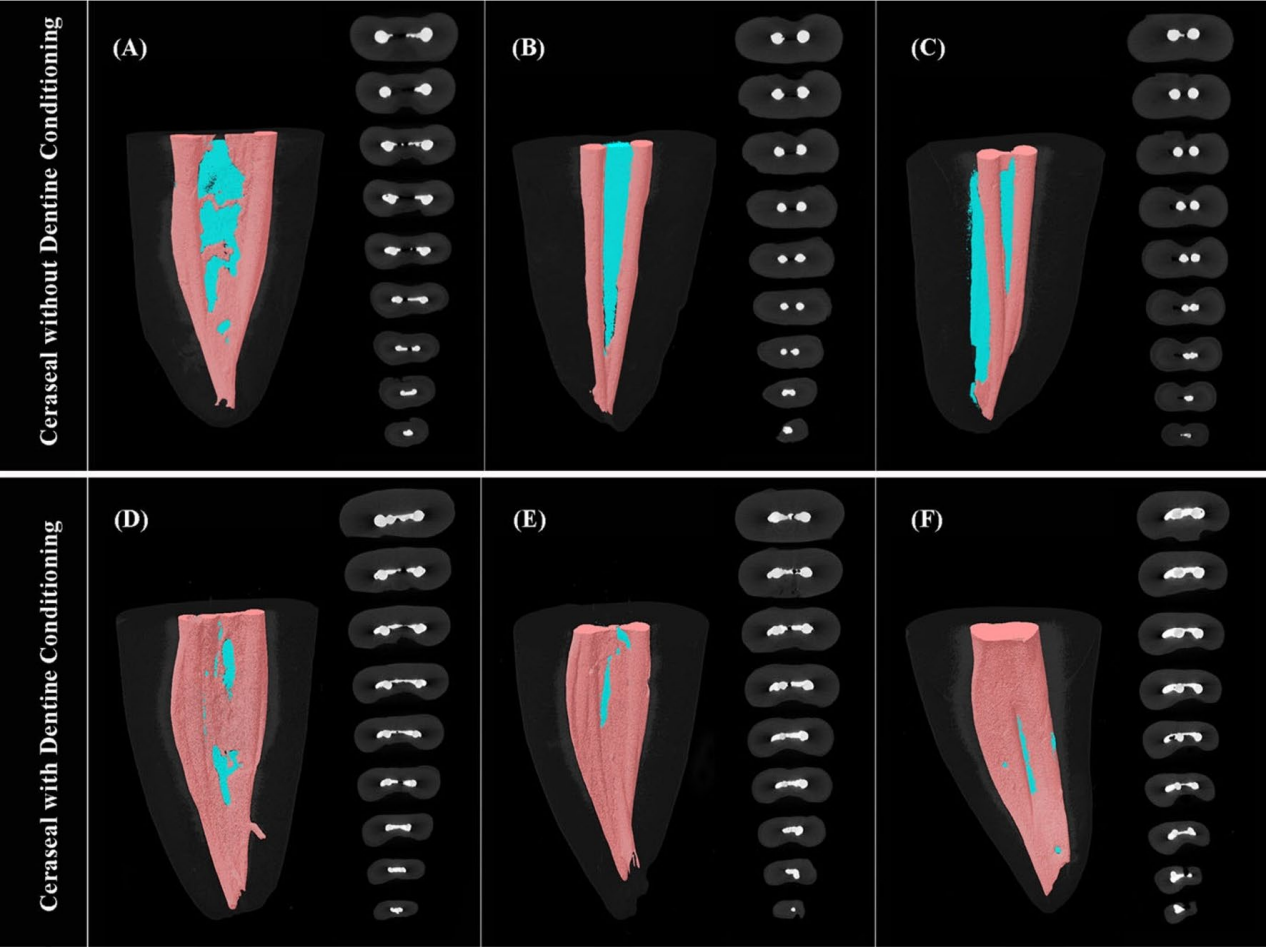
Three‐dimensional reconstructions of three representative mesial roots from control (A–C) and C‐HA‐treated groups (D–F), demonstrating obturation quality. Filling materials were marked in pink, with voids shown in blue. Corresponding two‐dimensional axial cross‐sections at coronal, middle and apical levels illustrated the material distribution using a grayscale scheme: gutta‐percha appears in grey, sealer in white and voids in black.

The 3D reconstructions presented in Figure 6 corroborated the quantitative findings, demonstrating significant differences in obturation quality between these two groups. Control specimens exhibited extensive void formation concentrated primarily within isthmus regions, whereas C‐HA‐treated roots showed minimal voids in these areas. Axial cross‐sectional analysis at coronal, middle and apical levels revealed significantly enhanced sealer penetration throughout isthmus spaces in the C‐HA group, with better adaptation observed in the cervical and middle thirds. Both groups showed satisfactory obturation quality in the main canal spaces, with minimal void formation observed in these regions.

### 3.2 SEM Evaluation

**FIGURE 7:**
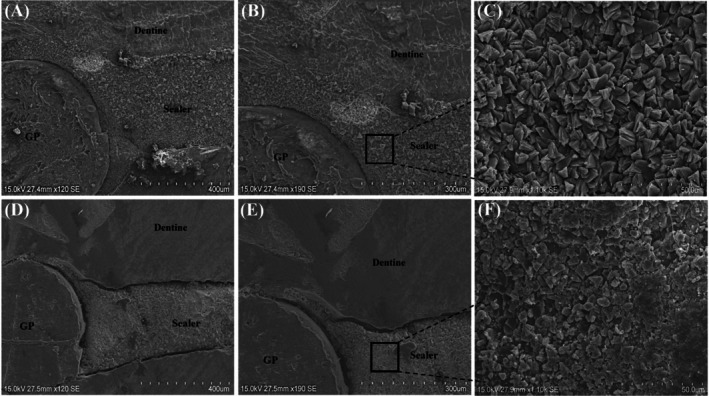
SEM images of sectioned root surfaces at 4 mm from the apex: (A–C) C‐HA treated group and (D–F) control group at increasing magnifications (A, D: ×120; B, E: ×190; C, F: ×1100). Images A, B and D, E demonstrated sealer adaptation to canal walls, while C and F showed the surface morphology of the sealer‐dentin interface.

SEM evaluation of the sealer‐dentin interface in the C‐HA group (Figure 7D,E), primarily examined at isthmus canal walls, demonstrated optimal integration characterised by a continuous, gap‐free transition zone, indicating the improvement of micromechanical interlocking. A homogeneously distributed hybrid layer was observed, suggesting effective penetration of the tricalcium silicate sealer into dentinal tubules due to C‐HA‐induced surface hydrophilicity. Besides, the gutta‐percha‐sealer interface also showed superior adaptation without visible gaps. Notably, the interface exhibited densely packed crystalline precipitates with trigonal pyramidal morphology (Figure 7F). This mineralization pattern aligned with the bioactive properties of C‐HA and contributed to reduced microleakage and enhanced long‐term bond durability.

In contrast, the control group exhibited discontinuous interfacial contact between dentine and sealer, with visible gaps at the dentin‐sealer interface and gutta‐percha‐sealer interface, which suggests incomplete sealer infiltration (Figure 7A,B). Unlike the C‐HA group, these samples lacked a clear hybrid layer and contained residual debris in the tubules. Precipitates in this group were sparse and globular (Figure 7C), reflecting less effective mineralization.

## 4 Discussion

The clinical challenge of endodontic irrigation lies in its paradoxical nature; while essential for effective disinfection, it simultaneously compromises the structural integrity of dentine. This study adopted the American Association of Endodontists (AAE)‐recommended irrigation protocol using 3% NaOCl followed by 17% EDTA (Baumgartner and Mader 1987). Though clinically, this protocol remains the gold standard for its well‐documented antimicrobial action and smear layer removal capacity (Zehnder et al. 2002; Hülsmann et al. 2003), it induces significant alterations in the dentine substrate. Specifically, NaOCl‐mediated collagen degradation and EDTA‐induced demineralization collectively reduce the surface free energy and wettability of dentine (Ferrari et al. 2004; Ballal et al. 2013; Gu et al. 2017). These physicochemical changes adversely affect sealer adaptation and tubular penetration, ultimately predisposing to interfacial gap formation and microleakage, which are the critical factors in treatment failure (Kishen 2006). These challenges are particularly significant in anatomically complex areas such as isthmus regions. Therefore, developing post‐irrigation strategies to restore the surface properties of dentine represents a promising approach to enhance clinical outcomes.

This study demonstrated that C‐HA dentine conditioning effectively enhanced single‐cone obturation quality, especially in isthmus regions. Previous research reported the capacity of C‐HA to strengthen dentine mechanical properties and enhance surface wettability, which promoted deeper sealer penetration into dentinal tubules (Hashmi, Sodhi, and Kishen 2019; Hashmi, Zhang, and Kishen 2019). Its specific impact on obturation quality in complex anatomy has not been investigated. Our work provided the first comprehensive assessment using both high‐resolution Micro‐CT (8.6 μm) and SEM analysis to evaluate obturation quality. Unlike destructive slide sectioning techniques that risk creating artificial voids or cracks during sample preparation, which potentially distort void percentage measurements, Micro‐CT preserves specimen integrity while providing complete volumetric assessment of the entire root canal system (Jung et al. 2005; Keleş et al. 2014; Wolf et al. 2014) Compared to conventional two‐dimensional imaging techniques, which are limited by structural superimposition for obscure anatomical details and compromise interpretation accuracy, high‐resolution Micro‐CT provides unobstructed visualisation of root canal anatomy and obturation qualities without the interpretive challenges of image overlap. Moreover, this technique achieves precise 11.2 μm resolution void detection (Orhan et al. 2018) while maintaining samples for subsequent SEM analysis. The combination of Micro‐CT with SEM examination offers a powerful analysis that simultaneously provides comprehensive three‐dimensional obturation assessment and detailed characterisation of dentin‐sealer bioactive interactions, thereby providing important investigations into the conditioning mechanism of C‐HA and its clinical benefits for bioceramic obturation.

The results revealed a significant spatial distribution of void formation, with the control group exhibiting approximately 20% total voids and 56% voids specifically within isthmus regions, which was consistent with a previous study that reported the limitations of single‐cone obturation in teeth with isthmus (Ma and Liang 2025). C‐HA treatment markedly improved obturation quality, achieving a 37% reduction in isthmus voids, with SEM analysis confirming superior interfacial adaptation characterised by continuous, gap‐free interfaces. These enhancements were mainly through three synergistic mechanisms, as reported before: (1) increased surface hydrophilicity that improved sealer flow into isthmus regions, (2) bioactive remineralisation via amorphous calcium phosphate nucleation and (3) collagen matrix reinforcement through intrafibrillar mineralisation (Hashmi, Sodhi, and Kishen 2019; Hashmi, Zhang, and Kishen 2019). The combination of these mechanisms enhanced sealer‐dentin contact in isthmus regions, suggesting the potential of C‐HA as a valuable adjunct for single‐cone obturation in complex root canal systems.

The differential void distribution across root canal levels provided valuable guidance for the future optimization of both the C‐HA nanocomplexes and the single‐cone technique. Although C‐HA treatment demonstrated significant improvements in cervical and middle thirds, apical regions showed comparable void percentages with the control group. This variation likely came from the anatomical differences in isthmus accessibility, as the wider dimension and greater surface area of coronal and middle isthmus regions facilitated more effective C‐HA penetration. Moreover, a previous study showed that approximately 35.2% of the isthmus volume was filled by residual hard tissue debris after instrumentation, which persisted through obturation (Endal et al. 2011). Given that chitosan nanoparticles in C‐HA nanocomplexes demonstrated smear layer removal efficacy comparable to 17% EDTA (Ratih et al. 2020), combined with their superior penetration capability compared to EDTA irrigant, might account for the significant void reduction in isthmus areas through effective debris dissolution.

Regarding apical regions, analysis of the entire root canal system revealed comparable void percentages in apical regions between C‐HA treatment (14%) and controls (15%). These values were consistent with reported outcomes for single‐cone technique obturation (Weis et al. 2004), indicating that although there was no improvement, C‐HA treatment maintained apical seal integrity to conventional methods without dentine conditioning. This observation might be attributed to two anatomical factors: first, sclerosis and debris in the apical isthmus region might restrict nanoparticle penetration (Paqué et al. 2006); second, the narrow isthmus and limited volume in the apical thirds might be sufficiently obturated by the hydraulic pressure generated during single‐cone placement (Teixeira et al. 2003), minimising the additional benefit of C‐HA conditioning.

SEM analysis demonstrated the bioactive potential of C‐HA through examination of root sections obtained 4 mm from the apex to minimise confounding variables from sclerotic dentine and anatomical complexities in the apical third (Kinney et al. 2005). Notably, dense trigonal pyramidal crystalline precipitates similar to the hydroxyapatite crystal were observed at the sealer‐dentin interface in the C‐HA group. Given the absence of phosphate in either Ceraseal or storage media, the phosphate from these crystalline structures might originate from the C‐HA, which corroborates the previous study (Hashmi, Zhang, and Kishen 2019). While conventional irrigation compromised collagen integrity and created hydrophobic dentine surfaces that impeded hydrophilic sealer penetration, C‐HA treatment formed a hydrophilic interfacial layer composed of chitosan‐hydroxyapatite nanocomplexes along canal walls. This layer, characterised by carboxyl groups from chitosan and interstitial water from amorphous calcium phosphate, significantly enhanced dentine wettability for tricalcium silicate sealers (Kalliola et al. 2017). Furthermore, the nanocomplexes likely penetrated the collagen matrix, occupying intrafibrillar spaces to promote biomimetic mineralization and restore the native collagen‐mineral coupling (Muzzarelli and Muzzarelli 2002; Chen et al. 2015), thereby reinforcing the interfacial matrix. Besides, an improvement in gutta‐percha‐sealer interfacial adaptation in the C‐HA treatment group compared to controls was noted. This enhanced adaptation likely results from the pre‐obturation conditioning protocol, wherein activation of the C‐HA solution using master cone gutta‐percha points facilitated the formation of a bioactive coating on the gutta‐percha surface. This coating, composed of chitosan‐hydroxyapatite complexes, substantially improved the surface wettability of the gutta‐percha point and promoted superior bonding with the bioceramic sealer. In contrast, control specimens treated with deionised water activation exhibited greater interfacial gap formation.

Several limitations must be considered when interpreting these findings. Although the sample size provided adequate power (80%) for detecting major differences in primary outcomes, it may be insufficient to reveal more minor differences, particularly in the challenging apical regions where anatomical complexity and sclerotic dentine are prevalent (Varoquaux 2018). Furthermore, despite the standardisation of tooth selection criteria (Vertucci Type II and Yin Type IV canals, curvature < 20°), inherent variations in dentine microstructure, tubular density and isthmus morphology among extracted teeth may introduce confounding variables. Also, procedural errors including occasional root perforations and transportation during instrumentation were observed, which could compromise the validity of the study's results. Moreover, this study mainly focuses on the Ceraseal sealer and single‐cone obturation technique; while providing controlled experimental conditions, it limits direct clinical extrapolation as the dentine conditioning did not combine with widely used bioceramic materials like iRoot sealer or alternative obturation techniques such as warm vertical compaction. Notably, AH Plus, the gold standard sealer, is not applicable in this study due to its reliance on dry dentine tubules for adhesion, which conflicts with our hydrated C‐HA conditioning protocol. Furthermore, the absence of comparative evaluation with other dentine conditioners, such as EDTA or chitosan alone, restricts a comprehensive assessment of C‐HA relative efficacy. Technical limitations should also be noted. For example, potential SEM vacuum‐induced artefacts from unembedded specimens may cause partial collapse of unmineralized collagen or artificial exaggeration of interfacial gaps, which may affect the SEM interpretations. Additionally, although the use of deionised water as a control is methodologically acceptable, its divergence from clinical saline use may reduce translational relevance. The fixed 5‐min C‐HA application time, though based on preliminary studies, also requires further optimization in future research. Finally, the study design did not evaluate critical clinical parameters, such as long‐term interfacial stability under cyclic loading or resistance to bacterial microleakage.

Despite these limitations, the current findings offer significant clinical implications for modern endodontic therapy. Future investigations should aim to standardise and optimise C‐HA conditioning protocols, including potential modifications such as sonic activation or ultrasonic activation to enhance apical penetration. Comparative studies with other conditioning agents would also help to indicate the relative efficacy of C‐HA conditioners. Furthermore, the incorporation of radiopaque markers into the C‐HA conditioner could ensure clinical visualisation of nanocomplex distribution. Overall, these results provide a robust scientific foundation for advancing C‐HA conditioner development and a clear direction for future research aimed at refining single‐cone obturation techniques.

## 5 Conclusion

C‐HA dentine conditioning significantly reduces void formation in the root canal system and isthmus regions, with remarkable improvements in obturation quality in coronal and middle thirds. Its bioactive properties enhance sealer‐dentin interfacial integrity through intrafibrillar remineralization. These findings highlight the potential of C‐HA as a critical adjunct to single‐cone techniques.

## Author Contributions

**Eissa Sameer Bunashi:** investigation, methodology, data curation, formal analysis, writing – original draft, writing – review and editing. **Mingxin Hu:** data curation, formal analysis, writing – original draft, writing – review and editing. **Angeline Hui Cheng Lee:** formal analysis, supervision, validation, writing – original draft, writing – review and editing. **Chengfei Zhang:** project administration, supervision, funding acquisition, writing – review and editing. **Anil Kishen:** resources, conceptualization, methodology, supervision. **Jeffrey Wen Wei Chang:** conceptualization, methodology, supervision, writing – review and editing, funding acquisition.

## Ethics Statement

The collection of extracted human teeth in this study has been approved by the Institutional Review Board of the University of Hong Kong/Hospital Authority Hong Kong West Cluster, IRB ref. Number: UW 20‐625.

## Conflicts of Interest

The authors declare no conflicts of interest.

## Supporting information


**Figure S1:** Two‐dimensional axial cross‐sections of three representative mesial roots from the control (A–C) and C‐HA treated (D–F) groups are shown, comparing pre‐ and post‐instrumentation root canal morphology. The left side displayed the root canal before instrumentation, while the right side illustrated the canal following instrumentation.

## Data Availability

The data that support the findings of this study are available from the corresponding author upon reasonable request.
