# Supplementary material for: Enhancing the Quality of Single Cone Obturation Using Hydroxyapatite Precursor Grafted Nanocomplex for Dentine Conditioning: An In Vitro Study
Source: Int Endod J. 2025 Sep 12;59(1):177–88. doi: 10.1111/iej.70034 (PMC12701747; doi:10.1111/iej.70034)
Supplement: Supplementary file 1 — Figure S1: Two‐dimensional axial cross‐sections of three representative mesial roots from the control (A–C) and C‐HA treated (D–F) groups are shown, comparing pre‐ and post‐instrumentation root canal morphology. The left side displayed the root canal before instrumentation, while the right side illustrated the canal following instrumentation. [file IEJ-59-177-s001.docx]

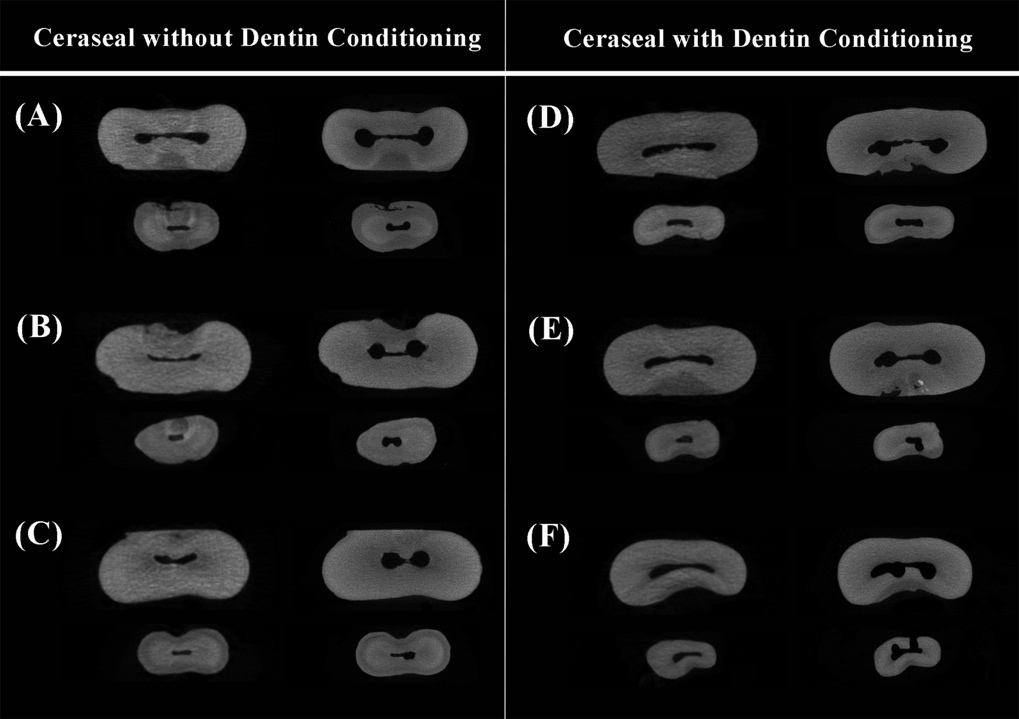


**FIGURE S1** Two-dimensional axial cross-sections of three representative mesial roots from the control (A–C) and C-HA treated (D–F) groups are shown, comparing pre- and post-instrumentation root canal morphology. The left side displayed the root canal before instrumentation, while the right side illustrated the canal following instrumentation.
